# Improvement of an in vitro drug selection method for generating transgenic *Plasmodium berghei* parasites

**DOI:** 10.1186/s12936-019-2851-6

**Published:** 2019-06-25

**Authors:** Akira Soga, Takahiro Shirozu, Mami Ko-ketsu, Shinya Fukumoto

**Affiliations:** National Research Center for Protozoan Diseases, Obihiro University of Agriculture and Veterinary Medicine, Inada-cho, Obihiro, Hokkaido 080-8555 Japan

**Keywords:** *Plasmodium berghei*, Transgenic parasite, Drug selection

## Abstract

**Background:**

Reverse genetics approaches have become powerful tools to dissect the biology of malaria parasites. In a previous study, development of an in vitro drug selection method for generating transgenic parasite of *Plasmodium berghei* was reported. Using this method, two novel and independent selection markers using the *P. berghei heat shock protein 70* promoter was previously established. While the approach permits the easy and flexible genetic manipulation of *P. berghei*, shortcomings include a low variety in promoter options to drive marker gene expression and increased complexity of the selection procedure. In this study, addressing these issues was attempted.

**Methods:**

To secure a variety of promoters, the use of a *P. berghei elongation factor*-*1α* promoter for marker gene expression was attempted. To simplify the procedure of in vitro selection, the establishment of a two cell-cycle culture method and its application for drug selection were attempted.

**Results:**

The *P. berghei elongation factor*-*1α* (*pbef*-*1α*) promoter, which is commonly used to drive marker gene expression, was successfully applied as an alternative promoter model for marker gene expression, using the parasite’s codon-optimized marker sequence. To simplify the in vitro selection method, a two cell-cycle culture method in which the merozoite was released by filtration of the culture containing matured schizont-infected erythrocytes was also developed and successfully applied for drug selection.

**Conclusion:**

The *pbef*-*1α* promoter was successfully applied in an in vitro selection system. The in vitro selection procedure also could be simplified for practical use using a two cell-cycle culture method. These improvements provide a more versatile platform for the genetic manipulation of *P. berghei.*

## Background

*Plasmodium berghei* is a useful in vivo rodent model of malaria. Its lifecycle can be reproduced in the laboratory and reverse genetics approaches have been established that have clarified much of its molecular biology [1–3]. The transfection efficacy of this parasite is low (10^−3^–10^−4^), therefore transfected parasites are usually selected by applying drug pressure [4]. As the parasite can’t be propagated in vitro [3, 4], in vivo drug selection systems have been used for generating transgenic parasites of *P. berghei.* As this has prevented the use of toxic drug-based selection systems for rodent hosts, only antifolate selection systems have been reported [3]. The limited number of available selection systems has hampered progressive genetic experiments such as multiple gene knockouts (KO) and KO rescues in a parasite [5–7]. To solve this problem, an in vitro drug selection method for *P. berghei* based on repeated short-term in vitro culture with a drug followed by parasite recovery in vivo have been developed [8]. This method enabled us to use toxic drug-based selection systems in rodent hosts. Using this in vitro selection method, puromycin-*N*-acetyltransferase (*pac*)-puromycin and blasticidin S deaminase (*bsd*)–blasticidin S (blasticidin) selection systems were successfully established for *P. berghei* [8, 9]. The triple sequential gene manipulation was also successfully demonstrated using these systems with a traditional pyrimethamine resistant *P. berghei* dihydrofolate reductase-thymidylate synthase (*pbdhfr*-*ts*)-pyrimethamine selection system [9]. Furthermore, in these in vitro selection systems, the target mutant parasite can be enriched to over 90% within 2 weeks [8, 9]. These results facilitated the isolation of the target gene-mutated parasite clones using fewer mice, shorter times, and lower costs in vivo [8, 9]. While the in vitro selection system has several advantages, there is still room for improvements in that approach as well.

Flexibility in the selection of a promoter sequence is important in *Plasmodium* research. However, in the in vitro selection method, only a *P. berghei heat shock protein 70* (*pbhsp70*) promoter was used to drive the expression of marker genes [8, 9]. The *pbhsp70* promoter is large (~ 1400 bp) and commonly used to drive strong expression of the target gene, such as a fluorescence marker, throughout the life cycle [10–12]. In some cases, over-expression of the marker gene causes unexpected effects for the parasite phenotype [13]. Therefore, it is desirable that an optimal choice of promoter should be possible for each assay. In some cases, the size of the targeting vector results in low recombination efficacy during gene manipulation. The choice of a most commonly used promoter *P. berghei elongation factor*-*1α* (*pbef*-*1α*, around 600 bp) for maker expression, can reduce the size of the targeting vector. Application of many kinds of available promoters for marker gene expression may facilitate useful future *Plasmodium* research.

The previous in vitro selection method for mutant parasite produced by homologous recombination method or *piggyBac* transposon-transposes recombination method had made it necessary to repeat the initial selection procedure, which was based on a single cell cycle of in vitro culture and in vivo parasite recovery, at least twice [8, 9]. Followed by drug selection, desired mutant parasite clone could be obtained by one round single cell cloning procedure. This repetition is however not optimal for practical use. With the present approach, it is possible to enrich the mutant parasite population to > 90% without repeating the selection procedure if two cell cycles of in vitro drug selection can be applied. In general, continuous in vitro culture of *P. berghei* is often difficult, for a variety of reasons. The organism cannot propagate in vitro because the merozoites cannot egress from the schizont without additional mechanical shear stress [3]. The low stability of the mouse RBC in vitro and a restricted ability to invade mature RBC is also a bottleneck in the long-term cultivation of *P. berghei* [14].

The objective of this study is to solve the problems described above for practical use of in vitro selection method. Firstly, to secure a variety of promoters, the application of the *pbef*-*1α* promoter, which is most commonly used to drive marker gene expression, was attempted in *P. berghei* as a model. Secondly, to simplify the procedure of drug selection, the establishment of a two cell-cycle culture method and its application for drug selection were attempted.

## Methods

### Experimental animals and parasites

ICR and BALB/c (5-week old) mice were obtained from CLEA Japan (Tokyo, Japan). The BALB/c mice were used for parasite cloning and the ICR mice were used for other experiments. *P. berghei* ANKA strain (obtained from Dr. M. Torii, Ehime University, Japan) was used [8].

### Codon optimization of marker genes

Codons of *pac* and *bsd* were optimized based on the *P. berghei* codon usage frequency from the Codon Usage Database (http://www.kazusa.or.jp/codon/), and the codons were changed to the most preferred codon without changing amino acid sequences. The codon-optimized markers (*pacPB* and *bsdPB*) were chemically synthesized by GenScript (Piscataway, NJ, USA).

### Plasmid construct

The *egfp* under control of the *pbhsp70* promoter and a terminator [8] was cloned into the plasmid pXL-BacII-DHFR (−) [8] (pXL/egfp). For pXL/hdhfr-pbef-1α-pac-egfp, *pac* and *hdhfr* under control of the *pbef*-*1α* promoter and *pbdhfr*-*ts* terminator was cloned into pXL/egfp. For pXL/ef-1α-pacPB-egfp and pXL/ef-1α-bsdPB-egfp, *pacPB* or *bsdPB* gene under control of the *pbef*-*1α* promoter and *pbdhfr*-*ts* terminator was cloned into pXL/egfp, respectively. The promoters and terminators were excised from a Yuda 2 plasmid (obtained from Dr. M. Yuda, Mie University). The pXL/hdhfr-pbef-1α-pac-egfp, pXL/pbef-1α-pacPB-egfp, and pXL/pbef-1α-bsdPB-egfp were transfected with the EGF-pgT [8]. For pBS/pbhsp70, a part of the *pbhsp70* gene was cloned into the pBluescript SK+ plasmid. The *pbef*-*1α* promoter was used in the same direction in all plasmids.

The correct sequences of all plasmid inserts were confirmed by DNA sequencing using an ABI PRISM 3100 Genetic Analyzer (Applied Biosystems, CA, USA).

### Parasite transfection and drug selection

All mutant parasites were generated using *piggyBac* transposon-transposase recombination methods. Parasite transfection experiments followed standard protocols basically as previously described [4]. Briefly, the schizonts purified by Nycodenz density gradient centrifugation were co-transfected with *piggyBac* vectors and transposase expression vector EGF-pgT [8] using Nucleofector 2b (Lonza, Basel, Switzerland) under the U-33 program. In vitro selection utilized puromycin and blasticidin except for the two cell-cycle culture method, which was performed as previously described [8, 9]. To generate mutant parasite clone containing *pac* driven by *pbef*-*1α* promoter (*pbef*-*1α*-*pac*) was performed using traditional in vivo pyrimethamine selection [4].

### In vitro drug selection using two cell-cycle culture

On day 1, 300 µl of infected *P. berghei* blood (parasitaemia 0.5–3.0%) was collected and placed in 5 ml of culture medium, centrifuged at 500×*g* for 8 min at room temperature (RT), and the supernatant was discarded. The blood was resuspended in 14.94 ml of culture medium. The suspension was placed into a 25 cm^2^ flask with 1.66 ml of puromycin stock solution (10 mg/ml in distilled water) to form a final concentration of 1.0 µg/ml and a total volume of 16.6 ml. This suspension was incubated for 20 h. On day 2, culture medium was removed after centrifugation of the culture at 500×*g* for 8 min. The pellet was resuspended in 14.94 ml of culture medium. The resuspended cultures were filtered using a filter unit with a 1.2 µm pore size and 32 mm diameter (Pall Corporation, Port Washington, NY, USA), attached to a 20 ml syringe. The filtrate containing free merozoite was put into a 25 cm^2^ flask, and quickly mixed with 300 μl of phenylhydrazine-treated non-infected mouse blood prepared by two washes in culture medium and centrifugation at 500×*g* for 8 min. The flask was placed on a shaker (NA-M101; Nissin, Tokyo, Japan) and incubated at about 40 rpm for approximately 4 h under the culture conditions described above. After incubation, 1.66 ml of puromycin solution were added into the flask to form a final concentration of 1.0 µg/ml and a total volume of 16.6 ml, which was then incubated for around 20 h. On day 3, the parasites were collected by centrifuging at 500×*g* for 5 min at RT, resuspended in about 100 μl of PBS, and injected intravenously into a naïve mouse.

### Mutant ratio calculation by microscopy and flow cytometry analysis

Parasites were identified with the Hoechst 33342 (Hoechst) stain and quantified using fluorescent microscopy as described previously [8]. The Hoechst-stained cells were analysed with a NovoCyte flow cytometer (ACEA Biosciences, San Diego, CA, USA) equipped with a 488 nm laser for eGFP and 405 nm laser for Hoechst. Parasites were gated using logarithmic forward/side scatter dot plots. The mutant ratio was analysed as previously described [8, 9]. At least 20,000 Hoechst-positive parasites were analysed. Data analysis was performed using the Novo Express program ver. 1.0.3 (ACEA Biosciences).

### Drug sensitivity test

Drug sensitivity tests were performed as previously described [8]. The infected *P. berghei* blood suspension was cultured with various concentrations of puromycin (*pbhsp70*-*pac* and *pbef*-*1α*-*pacPB*: 0–8 µg/ml, *pbef*-*1α*-*pac*: 0–4 µg/ml). IC_50_ values were calculated as previously described [8].

### Southern blot analysis

Southern blot analysis was performed as previously described [8]. The extracted genomic DNA (1.5 µg) were digested using *Nde*I. Digital chemiluminescence images were taken using an Ez-Capture MG (Atto, Tokyo, Japan).

### Real-time quantitative RT-PCR assays

Total RNA was isolated using the TRIzol reagent (Thermo Fisher Scientific), treated with RQ1 RNase-Free DNase (Promega, Fitchburg, WI, USA). cDNA was synthesized from 1.0 µg RNA using ReverTra Ace qPCR RT Kit (Toyobo, Osaka, Japan). The *pac*, *pacPB*, and *pbhsp70* standard curves were generated by tenfold serial dilution of pXL/hdhfr-pac-egfp [8], pXL/pbef-1α-pacPB-egfp, and pBS/pbhsp70 plasmid, respectively. Amplification and qPCR measurements were performed using the StepOne Real-Time PCR System, v 2.1 software (Applied Biosystems). All reactions were carried out using Fast SYBR Green Master Mix (Thermo Fisher Scientific). The qPCR cycle protocol was as follows: 20 s at 95 °C followed by 40 cycles of 3 s at 95 °C and 30 s at 60 °C. All samples were run in triplicate. *pbhsp70* was used as an endogenous control to normalize mRNA levels.

### Statistical analysis

Statistical analyses comparing each *pacPB*-integrated parasite clone against the wild type parasite were performed using two-tailed unpaired *t*-tests. The mutant ratio of each selection was compared using paired *t*-tests. Statistical analyses comparing IC_50_ values were performed using Dunnett’s multiple comparisons tests. All statistical analyses were performed using the GraphPad Prism program ver.5 (GraphPad, San Diego, CA, USA).

## Results

### *pbef*-*1α* promoter can be applied for in vitro puromycin selection using codon-optimized markers

**Fig. 1:**
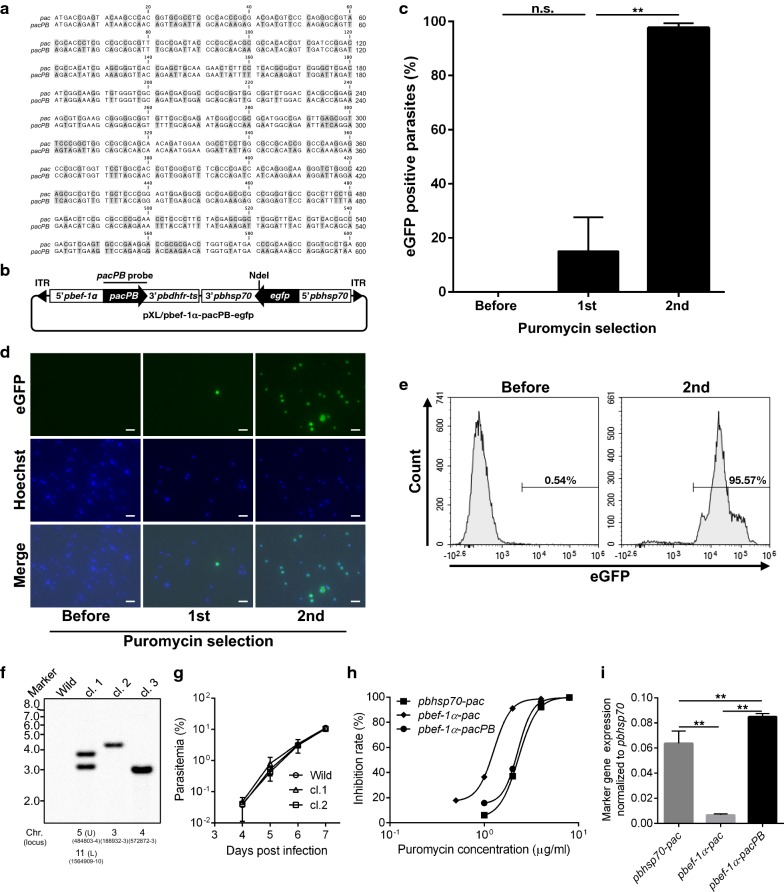
Generation of mutants using *pacPB* driven by *pbef*-*1α* promoter. **a** Nucleotide sequences of original (*pac*) and codon-optimized *pac* (*pacPB*). In the *pacPB* sequence, modified nucleotides are highlighted. **b** Schematic diagram of the *piggyBac* transposon vector containing *pacPB* and *egfp* expression cassettes (pXL/pbef-1α-pacPB-egfp). ITR: inverted terminal repeat. **c** The eGFP-positive parasite ratio after each puromycin selection. The ratio was analysed using fluorescence microscopy. Each bar represents the mean ± SD of four independent experiments. **p < 0.01, n.s.: not significant (paired *t*-tests). **d** Fluorescence images of parasites after each selection. Parasites were stained with Hoechst 33342. The scale bar represents 10 µm. **e** Flow cytometry analysis of the typical transfection line of eGFP positive mutant ratio after each puromycin selection. Numbers above the bracketed lines indicate the percentage of parasites with eGFP expression. **f** Southern blot analysis of three *pacPB* integrated mutant clones. Genomic DNA was digested using *Nde*I and hybridized with a *pacPB* probe. *Wild* wild type, *cl.* clone. Insertion sites of *piggyBac* transposon were shown blow. *U* upper band of cl.1, *L* lower band of cl.1, *Chr.* chromosome. **g** Growth assay of two *pacPB* integrated clones. Female ICR mice were infected intravenously with 1000 infected *P. berghei* erythrocytes. Parasitaemia of infected mice (Wild: n = 5, cl.1: n = 4, cl.2: n = 3) was monitored daily by examination of blood smears. *cl.* clone, *Wild* wild type. **h** Growth inhibition of pXL/hdhfr-pac-egfp [8]-transfected parasites (*pbhsp70*-*pac*), pXL/hdhfr-pbef-1α-pac-egfp-transfected parasites (*pbef*-*1α*-*pac*) obtained by pyrimethamine selection using *hdhfr* marker and pXL/pbef-1α-pacPB-egfp-transfected parasites (*pbef*-*1α*-*pacPB*) was evaluated by determining schizont development within a range of puromycin concentrations. **i** Real-time quantitative RT-PCR analysis of marker gene expression in *pbhsp70*-*pac, pbef*-*1α*-*pac, and pbef*-*1α*-*pacPB*. The value was normalized to the level of *pbhsp70* transcripts in each sample. **p < 0.01 (Dunnett’s multiple comparisons tests)

To investigate whether *pbef*-*1α* promoter can be used for mutant selection, wild type parasite was transfected with pXL/hdhfr-pbef-1α-pac-egfp containing *pac* driven by *pbef*-*1α* promoter, and then applied for puromycin selection. However, the desired eGFP-expressing mutants could not be detected using fluorescence microscopic analysis after the second selection. This result indicated that promoter activity might not have been sufficient for selection. To improve expression levels, codon usage for the *pacPB* sequence was optimized for the *P. berghei* genome (Fig. 1a), and a *pacPB* expression vector (pXL/pbef-1α-pacPB-egfp) was constructed (Fig. 1b). A wild type parasite was transfected with pXL/pbef-1α-pacPB-egfp. The in vitro puromycin selection was repeated twice as previously described [8] and the *egfp*-expressing mutant ratio was monitored before and after each selection. The ratios were 0.01 ± 0.01, 15.13 ± 12.51, and 97.65 ± 1.70% (mean ± SD) for each respective selection (Fig. 1c, d). A typical transfection line was also analysed using flow cytometry. More than 95% of the parasites expressed eGFP after the second selection (Fig. 1e). Three clones were isolated from the mutant parasite population selected in Fig. 1c by an in vivo method of limiting dilutions to confirm integration of *pacPB* into the genome. Southern blot analysis confirmed that *pacPB* was integrated into the genome. Two copies of *pacPB* were integrated into clone 1, and one copy of *pacPB* was integrated into clones. 2 and 3 (Fig. 1f). In clone 1 and 3, insertion position was on non-coding region. In clone 2, insertion position was on coding region of *PBANKA_0305500*. The growth of clones 1 and 2 was compared with that of wild type. The two clones grew equally as well as wild type parasites, which suggested that the integration of *pacPB* did not affect parasite growth (Fig. 1g). Then the effect of codon optimization was analysed. The IC_50_ values of three mutant clones containing *pac* driven by the *pbhsp70* promoter (*pbhsp70*-*pac*) [8] or *ef*-*1α* promoter (*pbef*-*1α*-*pac*), as well as *pacPB* driven by the *pbef*-*1α* promoter (*pbef*-*1α*-*pacPB*) were compared. *pbef*-*1α*-*pac* was generated by pyrimethamine selection. The IC_50_ values were 2.43 ± 0.29 µg/ml for *pbhsp70*-*pac*, 1.21 ± 0.19 µg/ml for *pbef*-*1α*-*pac*, and 2.12 ± 0.38 µg/ml for *pbef*-*1α*-*pacPB* (Fig. 1h). There was no significant difference between *pbhsp70*-*pac* and *pbef*-*1α*-*pacPB,* while the IC_50_ value of *pbef*-*1α*-*pacPB* was significantly increased compared to *pbef*-*1α*-*pac*. Real-time quantitative RT-PCR analysis also confirmed a significant increase in *pbef*-*1α*-*pacPB* expression (Fig. 1i). These results suggest that codon-optimization results in the improvement of *pac* expression. Thus, *pacPB* under the control of the *pbef*-*1α* promoter can be used as a positive selection marker in *P. berghei.*

### *pbef*-*1α* promoter can also be applied for in vitro blasticidin selection using codon-optimized marker

**Fig. 2:**
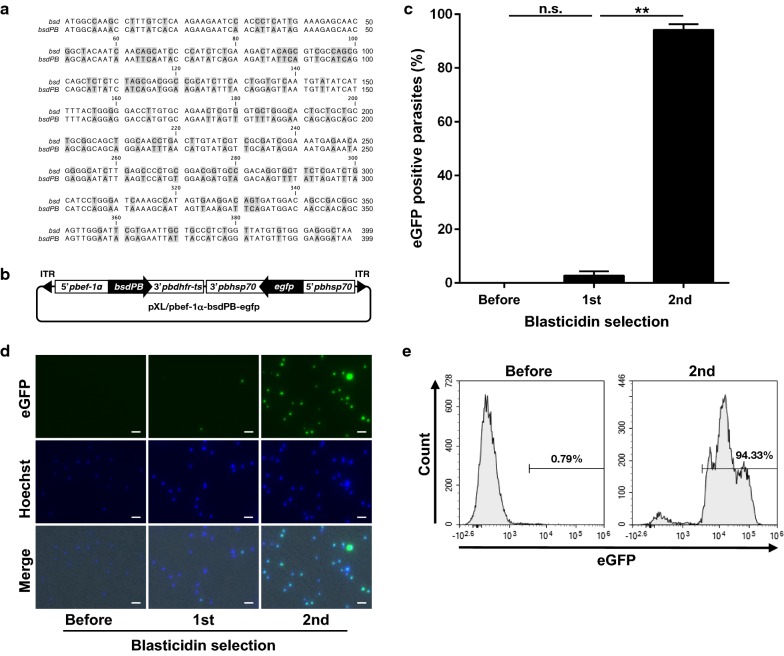
Generation of mutants using *bsdPB* driven by *pbef*-*1α* promoter. **a** Nucleotide sequences of original (*bsd*) and codon-optimized *bsd* (*bsdPB*). In the *bsdPB* sequence, modified nucleotides are highlighted. **b** Schematic diagram of the *piggyBac* transposon vector containing *bsdPB* and *egfp* expression cassettes (pXL/pbef-1α-bsdPB-egfp). *ITR* inverted terminal repeat. **c** The eGFP-positive parasite ratio after each blasticidin selection. The ratio was analysed using fluorescence microscopy. Each bar represents the mean ± SD of three independent experiments. **p < 0.01, n.s.: not significant (paired *t*-tests). **d** Fluorescence images of parasites after each selection. Parasites were stained with Hoechst 33342. The scale bar represents 10 µm. **e** Flow cytometry analysis of the typical transfection line of eGFP positive mutant ratio after each blasticidin selection. Numbers above the bracketed lines indicate the percentage of parasites with eGFP expression

To examine whether the *pbef*-*1α* promoter can be applied as a blasticidin selection system, a *bsdPB* sequence optimized for the codon usage of *P. berghei* was designed (Fig. 2a) and constructed the *bsdPB* expression vector (pXL/pbef-1α-bsdPB-egfp) (Fig. 2b). The in vitro selection procedure for the pXL/pbef-1α-bsdPB-egfp-transfected parasite was applied twice as previously described [9] and the *egfp*-expressing mutant ratio was monitored before and after each selection. The ratio was 0.01 ± 0.01, 2.81 ± 1.49, and 94.16 ± 2.15% (mean ± S.D.) after each selection (Fig. 2c, d). A typical transfection line was also analysed using flow cytometry. More than 94% of the parasites expressed eGFP after the second selection (Fig. 2e). Thus, *bsdPB* driven by the *pbef*-*1α* promoter can also be used as a positive selection marker in *P. berghei.*

### Development of improved in vitro selection method using two cell-cycle culture method

**Fig. 3:**
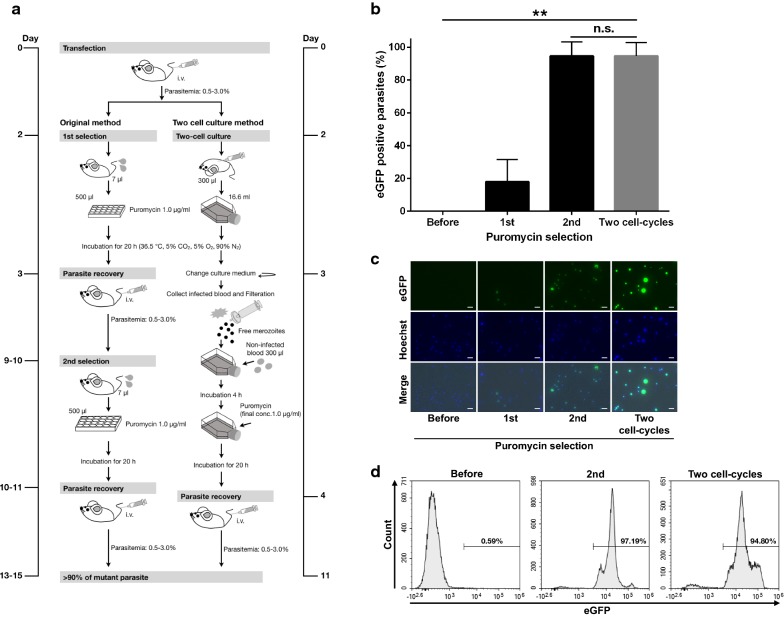
Establishment of two cell-cycle culture selection method. **a** Schematic diagram of original in vitro selection method and two cell-cycle culture selection method. **b** The eGFP-positive parasite ratio after each puromycin selection performed by original method and two cell-cycle culture method. The ratio was analysed using fluorescence microscopy. Each bar represents the mean ± SD of four independent experiments. **p < 0.01, n.s.: not significant (paired *t*-tests). **c** Fluorescence images of parasites after each selection and two cell-cycle culture selection. Parasites were stained with Hoechst 33342. The scale bar represents 10 µm. **d** Flow cytometry analysis of the typical transfection line of eGFP positive mutant ratio after each puromycin selection and two cell-cycle culture method. Numbers above the bracketed lines indicate the percentage of parasites with eGFP expression

To simplify the in vitro selection procedure, a method using two cell cycles in culture was examined whether the method could be applicable. A two cell-cycle in vitro culture procedure utilizing a filtration method was established (Fig. 3a) [15]. To determine its suitability as an in vitro selection method, it was used to select pXL/pbef-1α-pacPB-egfp-transfected parasites. After transfection, puromycin selection was performed by both the original method used in the previous study [8] and the two cell-cycle culture method. The *egfp*-expressing mutant ratio was examined before and after each selection for both methods. The ratio was 0.02 ± 0.03, 17.96 ± 13.51, 94.60 ± 8.66 and 94.59 ± 8.20% (mean ± SD) (Fig. 3b, c). The target mutant ratio after one selection using the two cell-cycle culture method was equal to that of the original method after a second selection. Flow cytometry analysis also confirmed that more than 94% of the parasites expressed eGFP after the two cell-cycle selection procedure (Fig. 3d). Thus, an in vitro puromycin selection method using a two cell-cycle culture method was successfully developed in *P. berghei.*

## Discussion

In this study, an improved in vitro drug selection method for generating transgenic parasites in *P. berghei* was described. Securing a variety of promoters and simplification of the selection procedure were attempted. In previous studies, the *pbhsp70* promoter was solely used to drive marker gene expression. In the present study, the *pbef*-*1α* promoter, most commonly used for marker gene expression, was successfully applied for in vitro selection by using a codon-optimized marker sequence. The *pbef*-*1α* promoter is bidirectional and its size (~ 600 bp) is smaller than *pbhsp70* (~ 1400 bp) [12]. This reduced size is an advantage, because large constructs often result in low recombination efficacy at the gene manipulation level as well as instability in *Escherichia coli* caused by the AT-rich *Plasmodium* genome [16]. The *pbhsp70* promoter is known to promote constitutive and strong expression of the gene throughout the life cycle [10]. Flexibility in the selection of a promoter is desirable to minimize any unexpected effects on the parasite phenotype after transfection. The use of the *pbef*-*1α* promoter as an in vitro selection method was, therefore, examined. In a *pac*-puromycin system, the mutant parasite with *pac* driven by the *pbef*-*1α* promoter could not be obtained. There were indications that the expression level of Pac under the *pbef*-*1α* promoter was insufficient for puromycin selection. The GC-richness (around 70%) of *pac* might affect the expression level of this marker gene in the AT-rich parasite genome [17]. To overcome this problem, codon usage within the *pac* sequence was optimized to that employed in the parasite genome. It has been reported that codon optimization of antibiotic genes results in the improvement of the drug selection efficacy in other organisms [18] as well as increased translation efficacy [19]. The *pbef*-*1α* promoter was successfully used with the codon-optimized *pacPB* for puromycin selection, suggesting that codon optimization increased the expression level of the marker gene. In fact, gene expression analysis by real-time RT PCR and IC_50_ value analysis showed a significantly increased expression level of the marker gene. As this codon optimization strategy could also be used in a blasticidin selection system, it has been suggested that a selection marker with GC-richness should be applied after sequence optimization for codon usage in malaria parasites [20–22].

In this study, an in vitro selection method using a two cell-cycle culture method was also successfully developed. In previous studies, established selection procedures based on in vitro culture and in vivo parasite recovery had to be applied twice [8, 9] as *P. berghei* merozoites cannot egress from the schizont in vitro without mechanical shear stress [3]. To simplify this procedure, a two cell-cycle culture method was applied as an in vitro selection system. The problem was addressed by applying a filtration protocol previously used for the purification of *Plasmodium yoelii* merozoites [15]. An in vitro selection procedure based on two cell-cycle culture with drug was successfully established. The desired mutant parasites were enriched to > 90% by a single in vitro selection. Overall selection efficacy was equivalent to that of the method used in the original in vitro selection [8]; however, the two cell-cycle culture approach shortened the total period required for generating mutant parasites. It took ~ 11 days to enrich the desired mutant parasite to > 90% using the two cell-cycle method, compared to ~ 14 days in the original method [8]. Therefore, the two cell-cycle method also permits rapid isolation of many mutant parasites. In addition, the two cell-cycle culture method requires only a single infected mouse for the procedure (Fig. 3a). The method’s high selection efficacy should allow us to isolate a clone using fewer than five mice, the number previously used for in vitro selection [8]. This method thus also has benefits for animal welfare.

## Conclusion

The *pbef*-*1α* promoter was successfully applied in an in vitro selection system. This resulted in an improved potential promoter variety for the in vitro selection system. Furthermore, the in vitro selection procedure could be simplified using the two cell-cycle culture method. This approach might minimize the time and animal usage often required in other in vitro selection systems. These improvements provide us a more flexible and user-friendly platform to perform genetic manipulation of *P. berghei.* Furthermore, the selection systems developed in this study could enable us to develop a genetically modified parasite-library, although further experiments are needed.

## Data Availability

All data generated or analysed during this study are included in this published article.

## Abbreviations

*pbef*-*1α*: *P. berghei elongation factor*-*1α*
*pbhsp70*: *P. berghei heat shock protein 70*
KO: gene knockouts
*Pac*: puromycin-*N*-acetyltransferase
*Bsd*: blasticidin S deaminase
blasticidin: blasticidin S
*pbdhfr*-*ts*: *P. berghei* dihydrofolate reductase-thymidylate synthase
*hdhfr*: human dihydrofolate reductase
RT: room temperature
